# Supplementary material for: Analogical reasoning in first and second languages
Source: PLoS One. 2025 Feb 11;20(2):e0318348. doi: 10.1371/journal.pone.0318348 (PMC11813118; doi:10.1371/journal.pone.0318348)
Supplement: S1 Appendix — (DOCX) [file pone.0318348.s001.docx]

S1 Appendix

Word Association Test

To create our stimuli, we conducted a word association test. Forty Japanese learners of English (20 males/20 females) participated in this task. Their ages varied from 18 to 31 (mean = 20.23 *SD* = 2.27). Stimuli were presented in English, and the participants were asked to write the first noun that came to their mind when they saw a stimulus word. Their English vocabulary size was measured by the Vocabulary Size Test [36]. Nation and Beglar [36] suggest that 8000 is the score that learners of English need to achieve to be capable of understanding a wide range of English materials. The mean score of their vocabulary size revealed that their English proficiency was at the intermediate level (mean = 7400, *SD* = 1942.21). As a rule, the most frequently listed words were chosen as alternatives. However, because we strived to avoid item repetition, in some cases, we selected the second or third most frequently listed words (e.g., *egg* was the most frequently listed word for omelet, but *tomato* was chosen to avoid repetition). When the equivalent number of participants listed different words for the same stimuli, a word with higher word frequency was chosen. Foreseeing that the same items were to be translated into Japanese for the corresponding Japanese experiment, phonologically similar words listed in the task such as *issue* for *tissue* were also avoided. If no appropriate word was found in the lists that participants produced, the first author chose a related word by consulting the Oxford English dictionary. Anomalous words were randomly chosen from the related words of A, which were assumed not to have a strong semantic relation with words B, C, or Answer. Overall, 700 words without an overlap were prepared. Strong semantic relations between words C and D were expected, as they share at least some similar concepts that can evoke possible relations; e.g., in hero : coward, they are antonyms as well as words to describe people. Similarly, words B’ and C’ were expected to have a strong semantic relation to their stimulus words B and C to serve as decent distractors. To understand the semantic distance between the stimulus words (words B and C) and related words such as distractors (B’, C’, Anom) and answers (D), the semantic distance of 500 pairs (100 each for BB’ BAnom, CC’, CD, CAnom) was obtained from the database of latent semantic analysis of Colorado University available online (http://lsa.colorado.edu/). One-way ANOVA revealed a significant main effect of pair type (*F*(4,488) = 67.61, *p*<.001). Pairwise comparisons with Bonferroni correction revealed a significant difference in semantic distance between CD and CAnom pairs, CC’ and CAnom pairs, and BB’ and BAnom pairs (*ps* < .001). Thus, words that were prepared as related words had a significantly closer relation with words B or C compared to the relation with anomalous words. The difference between CC’ and CD did not reach significance (*p*=.207). That means C’ words would be distracting enough against answers. There was a significant difference between CD and BB’ (*p*=.048), which may indicate that B’ words are not distracting enough compared to C’ words, but its effect size was relatively small (Cohen’s *d* = 0.32). Thus, related words prepared for C and B were semantically related to each stimulus word, and C words were semantically related to D. English words were prepared first and then translated into Japanese by the first and second authors. Then, the translation accuracy was checked by a Japanese professional translator.
