# Supplementary material for: Analogical reasoning in first and second languages
Source: PLoS One. 2025 Feb 11;20(2):e0318348. doi: 10.1371/journal.pone.0318348 (PMC11813118; doi:10.1371/journal.pone.0318348)
Supplement: S2 Appendix — It also contains tables from the final models with the effect of creativity as a fixed effect. (DOCX) [file pone.0318348.s002.docx]

**S2 Appendix**

*Plots of model results with difficulty of Experiment 1 (L1 Japanese).*


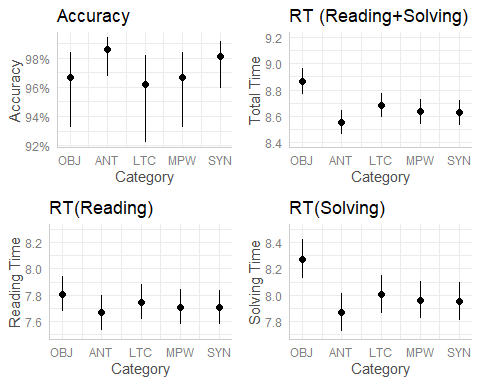


Mixed-effects model fitted to accuracy of L1 (Japanese) data with difficulty

| Fixed Effects | | | | | | |
| --- | --- | --- | --- | --- | --- | --- |
|  | Estimate | SE | 95% CI (2.5%) | 95% CI (97.5%) | z | p |
| (Intercept) | 2.823 | 0.371 | 2.097 | 3.549 | 7.618 | <.001 |
| Category(ANT) | 0.889 | 0.461 | -0.016 | 1.793 | 1.926 | 0.0541 |
| Category(LTC) | -0.141 | 0.412 | -0.948 | 0.665 | -0.344 | 0.7312 |
| Category(MPW) | 0.010 | 0.417 | -0.808 | 0.828 | 0.024 | 0.9807 |
| Category(SYN) | 0.584 | 0.425 | -0.250 | 1.418 | 1.372 | 0.1700 |
| Trial count | -0.183 | 0.105 | -0.388 | 0.022 | -1.751 | 0.0799 |
| Difficulty | -3.136 | 0.172 | -3.474 | -2.799 | -18.199 | <.001 |
| Random Effects | | | | | | |
|  | Variance | S.D. |  |  |  |  |
| Items(Intercept) | 1.138 | 1.067 |  |  |  |  |
| Participant(Intercept) | 1.900 | 1.378 |  |  |  |  |
| Paticipant\|Trial count (slope) | 0.207 | 0.455 |  |  |  |  |
| Participant\|Difficulty (slope) | 0.324 | 0.569 |  |  |  |  |
| Model fit | | | | | | |
| R² | Marginal | Conditional | AIC |  |  |  |
|  | 0.657 | 0.761 | 1656.900 |  |  |  |
|  |  |  |  |  |  |  |

*Note*. Sub category of problems were abbreviated as ANT(antonym) LTC (location-time context), MPW (material part-whole) and SYN (synonym). Trial count and difficulty were standardized.

Mixed-effects model fitted to accuracy of L1 (Japanese) data with creativity

| Fixed Effects | | | | | | |
| --- | --- | --- | --- | --- | --- | --- |
|  | Estimate | SE | 95% CI (2.5%) | 95% CI (97.5%) | z | p |
| (Intercept) | 1.542 | 0.300 | 0.954 | 2.130 | 5.141 | <.001 |
| Category(ANT) | 1.698 | 0.430 | 0.854 | 2.541 | 3.945 | <.001 |
| Category(LTC) | 0.388 | 0.395 | -0.386 | 1.163 | 0.982 | 0.326 |
| Category(MPW) | 0.809 | 0.397 | 0.031 | 1.587 | 2.038 | 0.042 |
| Category(SYN) | 1.035 | 0.397 | 0.256 | 1.814 | 2.605 | 0.009 |
| Trial count | -0.023 | 0.073 | -0.165 | 0.120 | -0.312 | 0.755 |
| Creativity | -1.346 | 0.109 | -1.560 | -1.132 | -12.313 | <.001 |
| Random Effects | | | | | | |
|  | Variance | S.D. |  |  |  |  |
| Items(Intercept) | 1.135 | 1.065 |  |  |  |  |
| Participant(Intercept) | 0.618 | 0.786 |  |  |  |  |
| Paticipant\|Trial count (slope) | 0.082 | 0.287 |  |  |  |  |
| Participant\|Creativity (slope) | 0.184 | 0.429 |  |  |  |  |
| Model fit | | | | | | |
| R² | Marginal | Conditional | AIC |  |  |  |
|  | 0.355 | 0.538 | 2529.450 |  |  |  |
|  |  |  |  |  |  |  |

*Note*. Sub category of problems were abbreviated as ANT(antonym) LTC (location-time context), MPW (material part-whole) and SYN (synonym). Trial count and creativity were standardized.

Mixed-effects model fitted to Total Time of L1 (Japanese) data with difficulty

| Fixed Effects | | | | | | |
| --- | --- | --- | --- | --- | --- | --- |
|  | Estimate | SE | 95% CI (2.5%) | 95% CI (97.5%) | t | p |
| (Intercept) | 8.995 | 0.049 | 8.899 | 9.091 | 183.900 | <.001 |
| Category(ANT) | -0.314 | 0.042 | -0.396 | -0.232 | -7.517 | <.001 |
| Category(LTC) | -0.186 | 0.042 | -0.267 | -0.104 | -4.472 | <.001 |
| Category(MPW) | -0.234 | 0.042 | -0.316 | -0.153 | -5.641 | <.001 |
| Category(SYN) | -0.242 | 0.041 | -0.323 | -0.161 | -5.853 | <.001 |
| Trial count | -0.063 | 0.008 | -0.078 | -0.048 | -8.207 | <.001 |
| Difficulty | 0.282 | 0.027 | 0.228 | 0.336 | 10.260 | <.001 |
| Random Effects | | | | | | |
|  | Variance | S.D. |  |  |  |  |
| Items(Intercept) | 0.011 | 0.106 |  |  |  |  |
| Participant(Intercept) | 0.060 | 0.246 |  |  |  |  |
| Paticipant\|Trial count (slope) | 0.001 | 0.030 |  |  |  |  |
| Paticipant\|Difficulty(slope) | 0.024 | 0.156 |  |  |  |  |
| Model fit | | | | | | |
| R² | Marginal | Conditional | AIC |  |  |  |
|  | 0.320 | 0.384 | 2795.360 |  |  |  |
|  |  |  |  |  |  |  |

*Note*. Sub category of problems were abbreviated as ANT(antonym) LTC (location-time context), MPW (material part-whole) and SYN (synonym). Trial count and difficulty were standardized.

Mixed-effects model fitted to Total Time of L1 (Japanese) data with creativity

| Fixed Effects | | | | | | |
| --- | --- | --- | --- | --- | --- | --- |
|  | Estimate | SE | 95% CI (2.5%) | 95% CI (97.5%) | t | p |
| (Intercept) | 8.972 | 0.049 | 8.877 | 9.068 | 184.042 | <.001 |
| Category(ANT) | -0.381 | 0.051 | -0.481 | -0.280 | -7.438 | <.001 |
| Category(LTC) | -0.218 | 0.051 | -0.318 | -0.118 | -4.278 | <.001 |
| Category(MPW) | -0.277 | 0.051 | -0.377 | -0.177 | -5.435 | <.001 |
| Category(SYN) | -0.281 | 0.051 | -0.380 | -0.182 | -5.551 | <.001 |
| Trial count | -0.073 | 0.008 | -0.089 | -0.058 | -9.352 | <.001 |
| Creativity | 0.158 | 0.019 | 0.121 | 0.196 | 8.287 | <.001 |
| Random Effects | | | | | | |
|  | Variance | S.D. |  |  |  |  |
| Items(Intercept) | 0.018 | 0.134 |  |  |  |  |
| Participant(Intercept) | 0.042 | 0.206 |  |  |  |  |
| Paticipant\|Trial count (slope) | 0.001 | 0.030 |  |  |  |  |
| Paticipant\|Creativity(slope) | 0.008 | 0.090 |  |  |  |  |
| Model fit | | | | | | |
| R² | Marginal | Conditional | AIC |  |  |  |
|  | 0.218 | 0.316 | 3186.014 |  |  |  |
|  |  |  |  |  |  |  |

*Note*. Sub category of problems were abbreviated as ANT(antonym) LTC (location-time context), MPW (material part-whole) and SYN (synonym). Trial count and creativity were standardized.

Mixed-effects model fitted to Reading Time of L1 (Japanese) data with difficulty

| Fixed Effects | | | | | | |
| --- | --- | --- | --- | --- | --- | --- |
|  | Estimate | SE | 95% CI (2.5%) | 95% CI (97.5%) | t | p |
| (Intercept) | 7.836 | 0.069 | 7.701 | 7.971 | 113.761 | <.001 |
| Category(ANT) | -0.143 | 0.032 | -0.207 | -0.080 | -4.445 | <.001 |
| Category(LTC) | -0.062 | 0.032 | -0.125 | 0.000 | -1.948 | 0.0536 |
| Category(MPW) | -0.100 | 0.032 | -0.162 | -0.037 | -3.119 | 0.0023 |
| Category(SYN) | -0.100 | 0.032 | -0.163 | -0.038 | -3.170 | 0.0019 |
| Trial count | -0.108 | 0.013 | -0.133 | -0.082 | -8.343 | <.001 |
| Difficulty | 0.061 | 0.016 | 0.029 | 0.094 | 3.731 | <.001 |
| Random Effects | | | | | | |
|  | Variance | S.D. |  |  |  |  |
| Items(Intercept) | 0.004 | 0.064 |  |  |  |  |
| Participant(Intercept) | 0.175 | 0.419 |  |  |  |  |
| Paticipant\|Trial count (slope) | 0.005 | 0.073 |  |  |  |  |
| Paticipant\|Difficulty(slope) | 0.004 | 0.064 |  |  |  |  |
| Model fit | | | | | | |
| R² | Marginal | Conditional | AIC |  |  |  |
|  | 0.108 | 0.171 | 3053.572 |  |  |  |
|  |  |  |  |  |  |  |

*Note*. Sub category of problems were abbreviated as ANT(antonym) LTC (location-time context), MPW (material part-whole) and SYN (synonym). Trial count and difficulty were standardized.

Mixed-effects model fitted to Reading Time of L1 (Japanese) data with creativity

| Fixed Effects | | | | | | |
| --- | --- | --- | --- | --- | --- | --- |
|  | Estimate | SE | 95% CI (2.5%) | 95% CI (97.5%) | t | p |
| (Intercept) | 7.833 | 0.069 | 7.697 | 7.969 | 112.813 | <.001 |
| Category(ANT) | -0.161 | 0.034 | -0.228 | -0.094 | -4.688 | <.001 |
| Category(LTC) | -0.074 | 0.034 | -0.141 | -0.007 | -2.180 | 0.0308 |
| Category(MPW) | -0.112 | 0.034 | -0.179 | -0.046 | -3.306 | 0.0012 |
| Category(SYN) | -0.112 | 0.033 | -0.178 | -0.047 | -3.358 | 0.0010 |
| Trial count | -0.109 | 0.013 | -0.135 | -0.083 | -8.251 | <.001 |
| Creativity | 0.031 | 0.014 | 0.002 | 0.059 | 2.116 | 0.0385 |
| Random Effects | | | | | | |
|  | Variance | S.D. |  |  |  |  |
| Items(Intercept) | 0.004 | 0.066 |  |  |  |  |
| Participant(Intercept) | 0.006 | 0.076 |  |  |  |  |
| Paticipant\|Trial count (slope) | 0.002 | 0.048 |  |  |  |  |
| Paticipant\|Creativity(slope) | 0.176 | 0.420 |  |  |  |  |
| Model fit | | | | | | |
| R² | Marginal | Conditional | AIC |  |  |  |
|  | 0.101 | 0.167 | 3081.741 |  |  |  |
|  |  |  |  |  |  |  |

*Note*. Sub category of problems were abbreviated as ANT(antonym) LTC (location-time context), MPW (material part-whole) and SYN (synonym). Trial count and creativity were standardized.

Mixed-effects model fitted to Solving Time of L1 (Japanese) data with difficulty

| Fixed Effects | | | | | | |
| --- | --- | --- | --- | --- | --- | --- |
|  | Estimate | SE | 95% CI (2.5%) | 95% CI (97.5%) | t | p |
| (Intercept) | 8.473 | 0.075 | 8.327 | 8.619 | 113.578 | <.001 |
| Category(ANT) | -0.412 | 0.066 | -0.542 | -0.283 | -6.231 | <.001 |
| Category(LTC) | -0.271 | 0.066 | -0.400 | -0.142 | -4.126 | <.001 |
| Category(MPW) | -0.315 | 0.066 | -0.444 | -0.186 | -4.787 | <.001 |
| Category(SYN) | -0.325 | 0.066 | -0.453 | -0.197 | -4.961 | <.001 |
| Trial count | -0.033 | 0.013 | -0.057 | -0.008 | -2.595 | 0.0131 |
| Difficulty | 0.433 | 0.046 | 0.343 | 0.522 | 9.456 | <.001 |
| Random Effects | | | | | | |
|  | Variance | S.D. |  |  |  |  |
| Items(Intercept) | 0.030 | 0.173 |  |  |  |  |
| Participant(Intercept) | 0.134 | 0.366 |  |  |  |  |
| Paticipant\|Trial count (slope) | 0.003 | 0.057 |  |  |  |  |
| Paticipant\|Difficulty(slope) | 0.071 | 0.267 |  |  |  |  |
| Model fit | | | | | | |
| R² | Marginal | Conditional | AIC |  |  |  |
|  | 0.301 | 0.382 | 5486.822 |  |  |  |
|  |  |  |  |  |  |  |

*Note*. Sub category of problems were abbreviated as ANT(antonym) LTC (location-time context), MPW (material part-whole) and SYN (synonym). Trial count and difficulty were standardized.

Mixed-effects model fitted to Solving Time of L1 (Japanese) data with creativity

| Fixed Effects | | | | | | |
| --- | --- | --- | --- | --- | --- | --- |
|  | Estimate | SE | 95% CI (2.5%) | 95% CI (97.5%) | t | p |
| (Intercept) | 8.446 | 0.075 | 8.298 | 8.594 | 112.070 | <.001 |
| Category(ANT) | -0.528 | 0.082 | -0.690 | -0.367 | -6.410 | <.001 |
| Category(LTC) | -0.332 | 0.082 | -0.492 | -0.171 | -4.041 | <.001 |
| Category(MPW) | -0.395 | 0.082 | -0.556 | -0.234 | -4.813 | <.001 |
| Category(SYN) | -0.398 | 0.082 | -0.558 | -0.238 | -4.877 | <.001 |
| Trial count | -0.051 | 0.012 | -0.074 | -0.027 | -4.263 | <.001 |
| Creativity | 0.231 | 0.031 | 0.169 | 0.293 | 7.338 | <.001 |
| Random Effects | | | | | | |
|  | Variance | S.D. |  |  |  |  |
| Items(Intercept) | 0.049 | 0.222 |  |  |  |  |
| Participant(Intercept) | 0.090 | 0.300 |  |  |  |  |
| Paticipant\|Trial count (slope) | 0.002 | 0.047 |  |  |  |  |
| Paticipant\|Creativity(slope) | 0.025 | 0.159 |  |  |  |  |
| Model fit | | | | | | |
| R² | Marginal | Conditional | AIC |  |  |  |
|  | 0.185 | 0.308 | 5906.312 |  |  |  |
|  |  |  |  |  |  |  |

*Note*. Sub category of problems were abbreviated as ANT(antonym) LTC (location-time context), MPW (material part-whole) and SYN (synonym). Trial count and creativity were standardized.

*Plots of model results with difficulty of Experiment 2 (L2 English).*


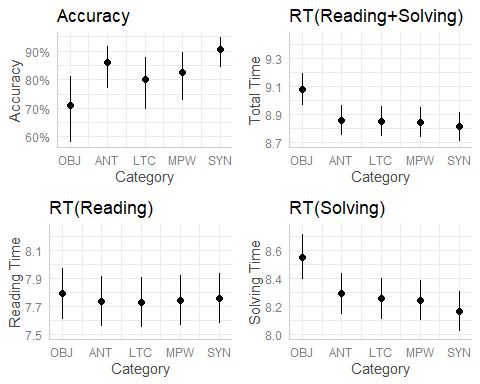


Mixed-effects model fitted to accuracy of L2 (English) data with difficulty

| Fixed Effects | | | | | | |
| --- | --- | --- | --- | --- | --- | --- |
|  | Estimate | SE | 95% CI (2.5%) | 95% CI (97.5%) | z | p |
| (Intercept) | 1.370 | 0.294 | 0.793 | 1.946 | 4.656 | <.001 |
| Category(ANT) | 0.908 | 0.356 | 0.211 | 1.605 | 2.553 | 0.0107 |
| Category(LTC) | 0.502 | 0.337 | -0.159 | 1.163 | 1.487 | 0.1369 |
| Category(MPW) | 0.660 | 0.347 | -0.020 | 1.340 | 1.901 | 0.0573 |
| Category(SYN) | 1.378 | 0.349 | 0.693 | 2.062 | 3.943 | <.001 |
| Trial count | -0.112 | 0.079 | -0.267 | 0.043 | -1.420 | 0.1555 |
| Difficulty | -3.114 | 0.204 | -3.514 | -2.713 | -15.243 | <.001 |
| Random Effects | | | | | | |
|  | Variance | S.D. |  |  |  |  |
| Items(Intercept) | 0.804 | 0.897 |  |  |  |  |
| Participant(Intercept) | 0.921 | 0.960 |  |  |  |  |
| Paticipant\|Trial count (slope) | 0.094 | 0.307 |  |  |  |  |
| Participant\|Difficulty (slope) | 1.020 | 1.010 |  |  |  |  |
| Model fit | | | | | | |
| R² | Marginal | Conditional | AIC |  |  |  |
|  | 0.690 | 0.805 | 2223.789 |  |  |  |
|  |  |  |  |  |  |  |

*Note*. Sub category of problems were abbreviated as ANT(antonym) LTC (location-time context), MPW (material part-whole) and SYN (synonym). Trial count and difficulty were standardized.

Mixed-effects model fitted to accuracy of L2 (English) data with creativity

| Fixed Effects | | | | | | |
| --- | --- | --- | --- | --- | --- | --- |
|  | Estimate | SE | 95% CI (2.5%) | 95% CI (97.5%) | z | p |
| (Intercept) | -0.019 | 0.253 | -0.515 | 0.477 | -0.076 | 0.9397 |
| Category(ANT) | 1.648 | 0.348 | 0.966 | 2.329 | 4.739 | <.001 |
| Category(LTC) | 0.824 | 0.336 | 0.165 | 1.483 | 2.451 | 0.0143 |
| Category(MPW) | 1.457 | 0.344 | 0.782 | 2.131 | 4.234 | <.001 |
| Category(SYN) | 1.834 | 0.344 | 1.159 | 2.509 | 5.325 | <.001 |
| Trial count | 0.040 | 0.046 | -0.051 | 0.130 | 0.853 | 0.3935 |
| Creativity | -0.951 | 0.056 | -1.061 | -0.841 | -16.935 | <.001 |
| Random Effects | | | | | | |
|  | Variance | S.D. |  |  |  |  |
| Items(Intercept) | 0.948 | 0.974 |  |  |  |  |
| Participant(Intercept) | 0.231 | 0.481 |  |  |  |  |
| Paticipant\|Trial count (slope) | 0.013 | 0.116 |  |  |  |  |
| Participant\|Creativity (slope) | 0.002 | 0.047 |  |  |  |  |
| Model fit | | | | | | |
| R² | Marginal | Conditional | AIC |  |  |  |
|  | 0.320 | 0.472 | 3676.668 |  |  |  |
|  |  |  |  |  |  |  |

*Note*. Sub category of problems were abbreviated as ANT(antonym) LTC (location-time context), MPW (material part-whole) and SYN (synonym). Trial count and creativity were standardized.

Mixed-effects model fitted to Total Time of L2 (English) data with difficulty

| Fixed Effects | | | | | | |
| --- | --- | --- | --- | --- | --- | --- |
|  | Estimate | SE | 95% CI (2.5%) | 95% CI (97.5%) | t | p |
| (Intercept) | 9.290 | 0.061 | 9.170 | 9.410 | 151.462 | <.001 |
| Category(ANT) | -0.227 | 0.045 | -0.314 | -0.140 | -5.091 | <.001 |
| Category(LTC) | -0.235 | 0.044 | -0.321 | -0.149 | -5.337 | <.001 |
| Category(MPW) | -0.240 | 0.044 | -0.326 | -0.155 | -5.516 | <.001 |
| Category(SYN) | -0.272 | 0.043 | -0.357 | -0.187 | -6.289 | <.001 |
| Trial count | -0.044 | 0.015 | -0.074 | -0.014 | -2.877 | 0.0068 |
| Difficulty | 0.203 | 0.030 | 0.145 | 0.262 | 6.834 | <.001 |
| FreqAveragePm_W1_log | -0.038 | 0.008 | -0.054 | -0.023 | -4.801 | <.001 |
| Random Effects | | | | | | |
|  | Variance | S.D. |  |  |  |  |
| Items(Intercept) | 0.009 | 0.093 |  |  |  |  |
| Participant(Intercept) | 0.087 | 0.295 |  |  |  |  |
| Paticipant\|Trial count (slope) | 0.007 | 0.084 |  |  |  |  |
| Paticipant\|Difficulty(slope) | 0.026 | 0.163 |  |  |  |  |
| Model fit | | | | | | |
| R² | Marginal | Conditional | AIC |  |  |  |
|  | 0.200 | 0.281 | 2731.460 |  |  |  |
|  |  |  |  |  |  |  |

*Note*. Sub category of problems were abbreviated as ANT(antonym) LTC (location-time context), MPW (material part-whole) and SYN (synonym). Trial count and difficulty were standardized.

Mixed-effects model fitted to Total Time of L2 (English) data with creativity

| Fixed Effects | | | | | | |
| --- | --- | --- | --- | --- | --- | --- |
|  | Estimate | SE | 95% CI (2.5%) | 95% CI (97.5%) | t | p |
| (Intercept) | 9.281 | 0.062 | 9.159 | 9.404 | 148.572 | <.001 |
| Category(ANT) | -0.262 | 0.050 | -0.360 | -0.164 | -5.229 | <.001 |
| Category(LTC) | -0.255 | 0.049 | -0.352 | -0.159 | -5.196 | <.001 |
| Category(MPW) | -0.269 | 0.049 | -0.365 | -0.173 | -5.492 | <.001 |
| Category(SYN) | -0.302 | 0.048 | -0.397 | -0.207 | -6.237 | <.001 |
| TrialCount_Difficulty_s | -0.056 | 0.016 | -0.087 | -0.025 | -3.551 | 0.0010 |
| Rating_Creativity_s | 0.104 | 0.017 | 0.070 | 0.137 | 6.077 | <.001 |
| FreqAveragePm_W1_log | -0.042 | 0.009 | -0.060 | -0.025 | -4.761 | <.001 |
| Random Effects | | | | | | |
|  | Variance | S.D. |  |  |  |  |
| Items(Intercept) | 0.012 | 0.110 |  |  |  |  |
| Participant(Intercept) | 0.075 | 0.274 |  |  |  |  |
| Paticipant\|Trial count (slope) | 0.008 | 0.088 |  |  |  |  |
| Paticipant\|Creativity(slope) | 0.005 | 0.072 |  |  |  |  |
| Model fit | | | | | | |
| R² | Marginal | Conditional | AIC |  |  |  |
|  | 0.238 | 0.140 | 2923.319 |  |  |  |
|  |  |  |  |  |  |  |

*Note*. Sub category of problems were abbreviated as ANT(antonym) LTC (location-time context), MPW (material part-whole) and SYN (synonym). Trial count and creativity were standardized.

Mixed-effects model fitted to Reading Time of L2 (English) data with difficulty

| Fixed Effects | | | | | | |
| --- | --- | --- | --- | --- | --- | --- |
|  | Estimate | SE | 95% CI (2.5%) | 95% CI (97.5%) | t | p |
| (Intercept) | 7.954 | 0.096 | 7.765 | 8.143 | 82.483 | <.001 |
| Category(ANT) | -0.057 | 0.038 | -0.131 | 0.018 | -1.494 | 0.1370 |
| Category(LTC) | -0.063 | 0.039 | -0.138 | 0.013 | -1.635 | 0.1038 |
| Category(MPW) | -0.050 | 0.038 | -0.123 | 0.024 | -1.331 | 0.1851 |
| Category(SYN) | -0.034 | 0.037 | -0.107 | 0.039 | -0.916 | 0.3609 |
| Trial count | -0.139 | 0.023 | -0.185 | -0.093 | -5.907 | <.001 |
| Difficulty | 0.044 | 0.018 | 0.009 | 0.079 | 2.435 | 0.0198 |
| FreqAveragePm_W1_log | -0.019 | 0.007 | -0.033 | -0.006 | -2.803 | 0.0062 |
| FreqAveragePm_W2_log | -0.023 | 0.006 | -0.036 | -0.010 | -3.583 | <.001 |
| Random Effects | | | | | | |
|  | Variance | S.D. |  |  |  |  |
| Items(Intercept) | 0.003 | 0.054 |  |  |  |  |
| Participant(Intercept) | 0.322 | 0.567 |  |  |  |  |
| Paticipant\|Trial count (slope) | 0.020 | 0.141 |  |  |  |  |
| Paticipant\|Dfficulty(slope) | 0.004 | 0.066 |  |  |  |  |
| Model fit | | | | | | |
| R² | Marginal | Conditional | AIC |  |  |  |
|  | 0.135 | 0.166 | 2929.343 |  |  |  |
|  |  |  |  |  |  |  |

*Note*. Sub category of problems were abbreviated as ANT(antonym) LTC (location-time context), MPW (material part-whole) and SYN (synonym). Trial count and difficulty were standardized.

Mixed-effects model fitted to Reading Time of L2 (English) data with creativity

| Fixed Effects | | | | | | |
| --- | --- | --- | --- | --- | --- | --- |
|  | Estimate | SE | 95% CI (2.5%) | 95% CI (97.5%) | t | p |
| (Intercept) | 7.955 | 0.097 | 7.765 | 8.145 | 82.179 | <.001 |
| Category(ANT) | -0.067 | 0.039 | -0.143 | 0.008 | -1.741 | 0.0834 |
| Category(LTC) | -0.070 | 0.039 | -0.147 | 0.007 | -1.793 | 0.0746 |
| Category(MPW) | -0.057 | 0.038 | -0.133 | 0.018 | -1.496 | 0.1364 |
| Category(SYN) | -0.044 | 0.038 | -0.118 | 0.030 | -1.172 | 0.2428 |
| Trial count | -0.141 | 0.023 | -0.187 | -0.095 | -6.024 | <.001 |
| Creativity | 0.024 | 0.016 | -0.008 | 0.055 | 1.477 | 0.1465 |
| FreqAveragePm_W1_log | -0.020 | 0.007 | -0.034 | -0.007 | -2.909 | 0.0046 |
| FreqAveragePm_W2_log | -0.024 | 0.006 | -0.037 | -0.011 | -3.679 | <.001 |
| Random Effects | | | | | | |
|  | Variance | S.D. |  |  |  |  |
| Items(Intercept) | 0.003 | 0.055 |  |  |  |  |
| Participant(Intercept) | 0.320 | 0.566 |  |  |  |  |
| Paticipant\|Trial count (slope) | 0.020 | 0.141 |  |  |  |  |
| Paticipant\|Creativity(slope) | 0.004 | 0.065 |  |  |  |  |
| Model fit | | | | | | |
| R² | Marginal | Conditional | AIC |  |  |  |
|  | 0.135 | 0.168 | 2932.160 |  |  |  |
|  |  |  |  |  |  |  |

*Note*. Sub category of problems were abbreviated as ANT(antonym) LTC (location-time context), MPW (material part-whole) and SYN (synonym). Trial count and creativity were standardized.

Mixed-effects model fitted to Solving Time of L2 (English) data with difficulty

| Fixed Effects | | | | | | |
| --- | --- | --- | --- | --- | --- | --- |
|  | Estimate | SE | 95% CI (2.5%) | 95% CI (97.5%) | t | p |
| (Intercept) | 8.814 | 0.086 | 8.648 | 8.980 | 103.009 | <.001 |
| Category(ANT) | -0.265 | 0.066 | -0.392 | -0.137 | -4.006 | <.001 |
| Category(LTC) | -0.299 | 0.065 | -0.425 | -0.172 | -4.578 | <.001 |
| Category(MPW) | -0.314 | 0.065 | -0.438 | -0.189 | -4.853 | <.001 |
| Category(SYN) | -0.393 | 0.064 | -0.516 | -0.269 | -6.122 | <.001 |
| Trial count | -0.001 | 0.025 | -0.052 | 0.049 | -0.047 | 0.9626 |
| Difficulty | 0.324 | 0.042 | 0.242 | 0.408 | 7.752 | <.001 |
| FreqAveragePm_W1_log | -0.039 | 0.012 | -0.061 | -0.016 | -3.329 | 0.0013 |
| Random Effects | | | | | | |
|  | Variance | S.D. |  |  |  |  |
| Items(Intercept) | 0.018 | 0.133 |  |  |  |  |
| Participant(Intercept) | 0.151 | 0.389 |  |  |  |  |
| Paticipant\|Trial count (slope) | 0.020 | 0.143 |  |  |  |  |
| Paticipant\|Dfficulty(slope) | 0.049 | 0.221 |  |  |  |  |
| Model fit | | | | | | |
| R² | Marginal | Conditional | AIC |  |  |  |
|  | 0.173 | 0.281 | 4957.704 |  |  |  |
|  |  |  |  |  |  |  |

*Note*. Sub category of problems were abbreviated as ANT(antonym) LTC (location-time context), MPW (material part-whole) and SYN (synonym). Trial count and difficulty were standardized.

Mixed-effects model fitted to Solving Time of L2 (English) data with creativity

| Fixed Effects | | | | | | |
| --- | --- | --- | --- | --- | --- | --- |
|  | Estimate | SE | 95% CI (2.5%) | 95% CI (97.5%) | t | p |
| (Intercept) | 8.804 | 0.090 | 8.628 | 8.979 | 97.436 | <.001 |
| Category(ANT) | -0.328 | 0.075 | -0.472 | -0.184 | -4.403 | <.001 |
| Category(LTC) | -0.341 | 0.073 | -0.482 | -0.199 | -4.659 | <.001 |
| Category(MPW) | -0.370 | 0.073 | -0.511 | -0.229 | -5.076 | <.001 |
| Category(SYN) | -0.451 | 0.072 | -0.590 | -0.311 | -6.254 | <.001 |
| Trial count | -0.016 | 0.025 | -0.065 | 0.032 | -0.663 | 0.5111 |
| Creativity | 0.150 | 0.027 | 0.098 | 0.206 | 5.623 | <.001 |
| FreqAveragePm_W1_log | -0.046 | 0.013 | -0.072 | -0.021 | -3.495 | <.001 |
| Random Effects | | | | | | |
|  | Variance | S.D. |  |  |  |  |
| Items(Intercept) | 0.025 | 0.160 |  |  |  |  |
| Participant(Intercept) | 0.145 | 0.381 |  |  |  |  |
| Paticipant\|Trial count (slope) | 0.019 | 0.137 |  |  |  |  |
| Paticipant\|Creativity(slope) | 0.013 | 0.116 |  |  |  |  |
| Model fit | | | | | | |
| R² | Marginal | Conditional | AIC |  |  |  |
|  | 0.173 | 0.281 | 4957.704 |  |  |  |
|  |  |  |  |  |  |  |

*Note*. Sub category of problems were abbreviated as ANT(antonym) LTC (location-time context), MPW (material part-whole) and SYN (synonym). Trial count and creativity were standardized.

Mixed-effects model fitted to accuracy of all data with creativity and an interaction of language and category

| Fixed Effects | | | | | | |
| --- | --- | --- | --- | --- | --- | --- |
|  | Estimate | SE | 95% CI (2.5%) | 95% CI (97.5%) | z | p |
| (Intercept) | 1.329 | 0.239 | 0.862 | 1.797 | 5.573 | <.001 |
| Category(ANT) | 2.021 | 0.358 | 1.319 | 2.722 | 5.648 | <.001 |
| Category(LTC) | 0.375 | 0.315 | -0.243 | 0.992 | 1.190 | 0.2340 |
| Category(MPW) | 0.937 | 0.323 | 0.304 | 1.570 | 2.903 | 0.0037 |
| Category(SYN) | 0.955 | 0.318 | 0.333 | 1.578 | 3.007 | 0.0026 |
| LANG(ENG) | -1.242 | 0.192 | -1.618 | -0.867 | -6.483 | <.001 |
| Trial count | 0.010 | 0.039 | -0.067 | 0.086 | 0.250 | 0.8025 |
| Creativity | -1.124 | 0.055 | -1.231 | -1.016 | -20.471 | <.001 |
| Category(ANT):LANG(ENG) | -0.510 | 0.260 | -1.018 | -0.001 | -1.963 | 0.0496 |
| Category(LTC):LANG(ENG) | 0.377 | 0.201 | -0.018 | 0.772 | 1.871 | 0.0614 |
| Category(MPW):LANG(ENG) | 0.359 | 0.214 | -0.060 | 0.779 | 1.678 | 0.0933 |
| Category(SYN):LANG(ENG) | 0.709 | 0.212 | 0.294 | 1.125 | 3.345 | 0.0008 |
| Random Effects | | | | | | |
|  | Variance | S.D. |  |  |  |  |
| Items(Intercept) | 0.72553 | 0.8518 |  |  |  |  |
| Participant(Intercept) | 0.35454 | 0.5954 |  |  |  |  |
| Paticipant\|Trial count (slope) | 0.03323 | 0.1823 |  |  |  |  |
| Participant\|Creativity (slope) | 0.06258 | 0.2502 |  |  |  |  |
| Model fit | | | | | | |
| R² | Marginal | Conditional | AIC |  |  |  |
|  | 0.405 | 0.52 | 6288.5 |  |  |  |
|  |  |  |  |  |  |  |

*Note*. Sub category of problems were abbreviated as ANT(antonym) LTC (location-time context), MPW (material part-whole) and SYN (synonym). Trial count and creativity were standardized.

Mixed-effects model fitted to Total Time of all data with creativity and an interaction of language and category

| Fixed Effects | | | | | | |
| --- | --- | --- | --- | --- | --- | --- |
|  | Estimate | SE | 95% CI (2.5%) | 95% CI (97.5%) | t | p |
| (Intercept) | 8.982 | 0.054 | 8.877 | 9.088 | 166.799 | <.001 |
| Category(ANT) | -0.338 | 0.048 | -0.433 | -0.243 | -6.986 | <.001 |
| Category(LTC) | -0.219 | 0.049 | -0.315 | -0.123 | -4.470 | <.001 |
| Category(MPW) | -0.254 | 0.047 | -0.346 | -0.161 | -5.353 | <.001 |
| Category(SYN) | -0.268 | 0.047 | -0.360 | -0.176 | -5.689 | <.001 |
| LANG(ENG) | 0.220 | 0.063 | 0.096 | 0.344 | 3.478 | <.001 |
| Trial count | -0.064 | 0.009 | -0.081 | -0.047 | -7.446 | <.001 |
| Creativity | 0.138 | 0.014 | 0.111 | 0.165 | 10.142 | <.001 |
| FreqAveragePm_W1_log | -0.015 | 0.006 | -0.028 | -0.003 | -2.377 | 0.0181 |
| Category(ANT):LANG(ENG) | 0.064 | 0.042 | -0.018 | 0.147 | 1.523 | 0.1279 |
| Category(LTC):LANG(ENG) | -0.022 | 0.043 | -0.107 | 0.063 | -0.510 | 0.6099 |
| Category(MPW):LANG(ENG) | -0.014 | 0.041 | -0.095 | 0.067 | -0.333 | 0.7389 |
| Category(SYN):LANG(ENG) | -0.038 | 0.041 | -0.118 | 0.041 | -0.941 | 0.3468 |
| Random Effects | | | | | | |
|  | Variance | S.D. |  |  |  |  |
| Items(Intercept) | 0.014 | 0.116 |  |  |  |  |
| Participant(Intercept) | 0.059 | 0.244 |  |  |  |  |
| Paticipant\|Trial count (slope) | 0.004 | 0.064 |  |  |  |  |
| Participant\|Creativity (slope) | 0.008 | 0.090 |  |  |  |  |
| Model fit | | | | | | |
| R² | Marginal | Conditional | AIC |  |  |  |
|  | 0.218 | 0.310 | 5831.796 |  |  |  |
|  |  |  |  |  |  |  |

*Note*. Sub category of problems were abbreviated as ANT(antonym) LTC (location-time context), MPW (material part-whole) and SYN (synonym). Trial count and creativity were standardized.

Mixed-effects model fitted to Reading Time of all data with creativity and an interaction of language and category

| Fixed Effects | | | | | | |
| --- | --- | --- | --- | --- | --- | --- |
|  | Estimate | SE | 95% CI (2.5%) | 95% CI (97.5%) | t | p |
| (Intercept) | 7.854 | 0.080 | 7.697 | 8.011 | 97.981 | <.001 |
| Category(ANT) | -0.127 | 0.033 | -0.192 | -0.062 | -3.832 | <.001 |
| Category(LTC) | -0.083 | 0.034 | -0.150 | -0.016 | -2.425 | 0.0161 |
| Category(MPW) | -0.090 | 0.032 | -0.154 | -0.027 | -2.794 | 0.0057 |
| Category(SYN) | -0.096 | 0.032 | -0.159 | -0.033 | -2.990 | 0.0031 |
| LANG(ENG) | 0.015 | 0.112 | -0.204 | 0.234 | 0.132 | 0.8950 |
| Trial Count | -0.126 | 0.013 | -0.152 | -0.100 | -9.471 | <.001 |
| Creativity | 0.029 | 0.008 | 0.012 | 0.045 | 3.407 | <.001 |
| FreqAveragePm_W1_log | -0.016 | 0.005 | -0.025 | -0.008 | -3.617 | <.001 |
| Category(ANT):LANG(ENG) | 0.046 | 0.038 | -0.029 | 0.121 | 1.199 | 0.2306 |
| Category(LTC):LANG(ENG) | 0.001 | 0.040 | -0.078 | 0.079 | 0.019 | 0.9848 |
| Category(MPW):LANG(ENG) | 0.032 | 0.038 | -0.042 | 0.107 | 0.856 | 0.3922 |
| Category(SYN):LANG(ENG) | 0.063 | 0.038 | -0.011 | 0.137 | 1.657 | 0.0975 |
| Random Effects | | | | | | |
|  | Variance | S.D. |  |  |  |  |
| Items(Intercept) | 0.003 | 0.057 |  |  |  |  |
| Participant(Intercept) | 0.239 | 0.488 |  |  |  |  |
| Paticipant\|Trial count (slope) | 0.012 | 0.112 |  |  |  |  |
| Model fit | | | | | | |
| R² | Marginal | Conditional | AIC |  |  |  |
|  | 0.108 | 0.197 | 5783.021 |  |  |  |
|  |  |  |  |  |  |  |

*Note*. Sub category of problems were abbreviated as ANT(antonym) LTC (location-time context), MPW (material part-whole) and SYN (synonym). Trial count and creativity were standardized.

Mixed-effects model fitted to Solving Time of all data with creativity and an interaction of language and category

| Fixed Effects | | | | | | |
| --- | --- | --- | --- | --- | --- | --- |
|  | Estimate | SE | 95% CI (2.5%) | 95% CI (97.5%) | t | p |
| (Intercept) | 8.426 | 0.074 | 8.281 | 8.571 | 113.810 | <.001 |
| Category(ANT) | -0.509 | 0.071 | -0.649 | -0.370 | -7.159 | <.001 |
| Category(LTC) | -0.322 | 0.071 | -0.461 | -0.183 | -4.540 | <.001 |
| Category(MPW) | -0.373 | 0.071 | -0.512 | -0.235 | -5.272 | <.001 |
| Category(SYN) | -0.390 | 0.070 | -0.528 | -0.252 | -5.542 | <.001 |
| LANG(ENG) | 0.258 | 0.090 | 0.081 | 0.435 | 2.856 | 0.0049 |
| Trial count | -0.036 | 0.013 | -0.062 | -0.010 | -2.680 | 0.0091 |
| Creativity | 0.209 | 0.021 | 0.168 | 0.251 | 9.823 | <.001 |
| Category(ANT):LANG(ENG) | 0.152 | 0.062 | 0.030 | 0.274 | 2.448 | 0.0144 |
| Category(LTC):LANG(ENG) | -0.006 | 0.062 | -0.128 | 0.116 | -0.092 | 0.9269 |
| Category(MPW):LANG(ENG) | -0.009 | 0.062 | -0.130 | 0.112 | -0.148 | 0.8821 |
| Category(SYN):LANG(ENG) | -0.083 | 0.061 | -0.202 | 0.036 | -1.370 | 0.1707 |
| Random Effects | | | | | | |
|  | Variance | S.D. |  |  |  |  |
| Items(Intercept) | 0.032 | 0.180 |  |  |  |  |
| Participant(Intercept) | 0.118 | 0.343 |  |  |  |  |
| Paticipant\|Trial count (slope) | 0.010 | 0.100 |  |  |  |  |
| Paticipant\|Creativity(slope) | 0.022 | 0.147 |  |  |  |  |
| Model fit | | | | | | |
| R² | Marginal | Conditional | AIC |  |  |  |
|  | 0.183 | 0.286 | 11126.060 |  |  |  |
|  |  |  |  |  |  |  |

*Note*. Sub category of problems were abbreviated as ANT(antonym) LTC (location-time context), MPW (material part-whole) and SYN (synonym). Trial count and creativity were standardized.
