# Supplementary material for: Analogical reasoning in first and second languages
Source: PLoS One. 2025 Feb 11;20(2):e0318348. doi: 10.1371/journal.pone.0318348 (PMC11813118; doi:10.1371/journal.pone.0318348)
Supplement: Attachment — Submitted filename: Response to Reviewers.docx [file pone.0318348.s003.docx]

Please take particular care to the comments raised by the reviewers, in particular:

- The justification, objectives and motivations for this research work should be articulated more.

- The methodology and its background would require further, deeper investigation. The links with the related literature should be more explored as well, highlighting the novelty of this paper. Be sure to cite the latest and more related references in the literature.

- Explain better and motivate the employed dataset and features, in particular the choice of the used data sample size.

- The reported computational analysis and discussion show to be vague and not robust enough, they should be articulated more.

- The manuscript structure needs also a substantial rearrangement, as outlined by the reviewers. Overall readability and presentation should be improved as well.

**Thank you for having given us the chance to improve our manuscript. As shown below, we responded to all the comments of the reviewers. We believe that the manuscript became shorter (13567 words originally and 12663 words this time) and more convincing. Also, we cited more latest articles in our manuscript.**

[8] Kapetanakis C, Conflitti S, Abdo S, Wright LK, Newman DL. (2024) Constructing analogies: Developing critical thinking through a collaborative task. *Biochemistry Molecular Biology Education*, *52*(5), 569–579. <https://doi.org/10.1002/bmb.21843>

[13] Israel, N. (2019). *The relationship between analogical problem solving and analogical reasoning.* [Doctoral dissertation, University of the Witwatersrand, Faculty of Humanities]. <https://wiredspace.wits.ac.za/server/api/core/bitstreams/59a73e0a-cc85-4f2b-ae8f-9fa767c26d12/content>

[21] Kucwaj, H., Ociepka, M., & Chuderski, A. (2022). Various sources of distraction during analogical reasoning. Memory & Cognition, 50(7), 1614-1628. <https://doi.org/10.3758/s13421-022-01285-3>

[67] Gray, M. E., & Holyoak, K. J. (2021). Teaching by analogy: From theory to practice. Mind, Brain, and Education, 15(3), 250–263. <https://doi.org/10.1111/mbe.12288>

**We considered other aspects suggested following the reviewers’ comments below.**

Reviewer #1: Great manuscript but incredibly lengthy. Would recommend this be broken up into at least two manuscripts, or at the very least have clearly defined sections. For example, the methods include results and results include methods and discussion items. This makes it difficult to follow the content of this analysis. As an aside, there should be continuous numbering throughout.

>>Introduction - would make this significantly more succinct. Line 6-7: "Foot tapping". This can be omitted, as it does not appear to contribute anything to the introduction.

**As suggested, to make the manuscript easier to read, we removed the line “Foot tapping was assigned as a concurrent task to the L1 analog group to reduce cognitive resources” on page 4.**

>>Methods - would remove all results (e.g lines 15-19). Statistical methods should be outlined in the methods section, not in the results section (e.g., lines 18-24, 1-17)).

**We removed the explanation of the word association test and put it in the appendix.**

>>Results - only report results, not items for the discussion (e.g.,lines 22-23 "this issue will be further discussed in the general discussion, recalling Hayakawa and Keysar’s study [22]). This is not necessary.

**We do agree with the reviewer that presenting Result section and Discussion section separately has been the norm in experimental psychology. However, given the complexity of the multivariate analyses, we opted for providing interpretations right after presenting the result to help readers follow our discussion. We believe this is not an uncommon approach. Some relatively recent articles such as Errington et al. (2022) and Flusberg et al. (2024) published in PLOS ONE also combined results and discussion sections.**

Errington, P. J., Thye, M., & Mirman, D. (2022). Difficulty and pleasure in the comprehension of verb-based metaphor sentences: A behavioral study. *PLOS ONE*, *17*(2), e0263781. <https://doi.org/10.1371/journal.pone.0263781>

Flusberg, S. J., Mackey, A., & Semino, E. (2024). Seatbelts and raincoats, or banks and castles: Investigating the impact of vaccine metaphors. *PLOS ONE*, *19*(1), e0294739. <https://doi.org/10.1371/journal.pone.0294739>

Reviewer #2: This is a timely and useful study that provides important predictors for analogical reasoning in bilinguals. It focuses on how analogies are processed in a second language and makes use of linguistic predictors like word frequencies, difficulty and creativity problems to show how they contribute to analogical reasoning. The study portrays how these predictors are useful in influencing performance of individuals’ analogical reasoning in first and second languages. The findings show among others that using a less familiar language hinders efficacy of analogical reasoning. Similarly, first language elicited higher accuracies and faster response time than the second language. This expands our understanding of how analogical reasoning is less detailed or effective when a second less familiar language is introduced into the equation. While this study is useful in many respects and to a large extent well executed, it can be improved to make it more robust. The following are observations and suggestions for improvement:

**Thank you for your careful reading and positive feedback. I hope you will find the revised version more convincing.**

>>1. There is an over use of examples in the background of the write up: the mitochondria analogy and the ray problem for instance are useful but have been repeated rendering them redundant without advancing the argument. It will be better to introduce them once and refer to them without explaining them in detail each time.

**Considering the length of the manuscript, we removed the explanation about Ray problems and Lightbulb problems and added them in the notes. Also, we revised the entire section on page 2.**

**Also, we removed “A cloud is like a sponge” metaphor as an example from page 4 and revised the paragraph about similarities in analogical reasoning.**

>>2. Some terms have been introduced without sufficient clarification. Examples include ‘relational similarity’ and ‘perceptual similarity’. These are important arguments that necessitate the study, yet they are only briefly explained. A detailed explanation is needed. Also, phrases like ‘analogical transfer is language-free’ needs to be in context. Without this contextualization it is not clear as proficiency differences can also play a role.

**To increase the readability, we rephrased the sentences on page 3 that introduced the term language-free.**

**We added an explanation of perceptual and relation similarities in analogical reasoning in the notes.**

>>3. The study makes reference to previous studies but does not really critically engage with them. This can be resolved by looking at their findings, contradictions and examining potential reasons for these outcomes in relation to the present study.

**We revised the entire section on page 2. Also, we added a paragraph to highlight the difference between the previous studies and the present study on page 3.**

**(p.3) These prior studies focused on the accuracy of problem solving in L2, rather than investigating the underlying mechanisms of the reasoning process itself. In analogical reasoning, knowledge transfer between domains occurs based on similarities [3]. However, how this similarity information is transferred in L2 remains unexplored. In addition, simply investigating the accuracy of analogical reasoning is not sufficient to fully understand at what point the differences between L1 and L2 reasoning processes occur.**

>>4. It will be beneficial to explain why reaction times are important to the study as the research questions place strong emphasis on this. For instance, how does reaction time contribute to understanding the cognitive processes behind analogical reasoning and why this is an important metric.

**To clarify the reasons to measure reaction times, we added an explanation on page 6**

**(p.6) In addition to accuracy, to investigate the time course of analogical reasoning in L2, this study analyzed three types of reaction times: the time to read analogies (e.g., delight : happiness :: woman : ???), the time to choose an answer (e.g., lady) from multiple choices (e.g., lady, man, success and TV), and the combined time for both. The reaction times were analyzed to investigate at what point processing differences occur in analogical reasoning in a less familiar language (L2).**

>>5. In the hypothesis, there is a focus on differences in accuracy and reaction time between L1 an L2 but this lacks specificity. For instance, there is the hypothesis that ‘lower accuracy is expected for the L2 condition’. This could be made more precise by stating the expected differences between specific relational times or levels of proficiency.

**We added reasoning to present our hypotheses.**

**The hypothesis is based on the previous studies as we stated**

**(p.6) The previous studies suggested that the use of a foreign (unfamiliar) language would result in lower accuracy for analogical reasoning [10, 14]. As in the previous study, lower accuracy is expected for the L2 condition. This is because less efficient language processing in L2 requires great cognitive load, leading to limited cognitive resources to handle analogical problems.**

**The interaction between languages (L1/L2) and similarities are explained in the third hypothesis.**

**(p.7)** **For instance, Jones et al. [27] reported higher accuracies for categorical analogies (e.g., hammer:tool) compared to compositional and causal analogies (e.g., allergy:sneeze). Therefore, assuming that the higher-level conceptual representations are comparable between L1 and L2 participants, it is possible that the accuracy for synonym analogies would be higher in both L1 and L2 conditions, while the accuracy between languages would vary for other categories. This hypothesis was tested by directly comparing the L1 and L2 conditions, with a specific focus on examining the interaction between problem categories and languages.**

>>6. Cognitive load associated with L2 processing is mentioned but not discussed in depth. It appears that cognitive load is an important factor in understanding differences in L1 and L2 performance and so a detailed discussion of how it influences analogical reasoning could strengthen the argument.

**We analyzed the effects of word frequency to investigate the cognitive load associated with L2 use in reasoning. This is because the higher the word frequency, the faster people process the words. Assuming people are less exposed to their L2 than their L1, it is expected that the effects of word frequency are greater in L2 than in L1. The inefficiency in lexical access would explain a cause of cognitive load in terms of L2 analogical reasoning. To highlight that point, we added the explanation below on page 8.**

**(p.8) In general, the higher the word frequency, the faster people process the words [28-32]. This is because the more frequently people encounter a word, the more easily they can access its lexical information. Assuming people are less exposed to their L2 than their L1, it is expected that the effects of word frequency are greater in L2 than in L1, as found in previous studies [28-32]. Additionally, low-frequency words are expected to hinder efficient analogical transfer to a greater extent, especially when solving problems. For instance, filament is a relatively low-frequency word that appeared in a story used in [14]…**

>>7. Take note of in-text citations. For instance, some citations have page numbers for direct quotes but others do not. There should be consistency.

**Thank you for pointing it out. We revised our manuscript checking in-text citations.**

>>8. In terms of the methodology, how were variables such as participants’ environment and potential distractions controlled for during tasks. These could affect time and accuracy.

**Our experiments were conducted online. Therefore, the experimental environments for different participants were unfortunately not well-controlled. This is an unfortunate consequence of online experiments. At this moment, however, there is no evidence to suggest that the unexpected noise was greater for one group than the other. At least, we did the following to minimize the noise.**

- **We asked all participants to use a laptop/desktop computer rather than a smartphone.**
- **We provided feedback to participants during the experiment to motivate them to concentrate on the experiments.**

**We agree with the reviewer that this is something to worry about. However, because even the long-beloved lab experiments, too, are subject to unexpected noise (i.e., lab effect), we are not here stating that the results of our online experiments were negatively affected by the uncontrolled environment. We are, in fact, currently investigating the potential impact of online experiments in a separate study. We would like to leave it to future research.**

>>9. The study emphasizes the importance of word frequency and what it does for L2 analogical reasoning. However, it missed explaining how specific frequency thresholds like high versus low frequency were determined. If this is available, it is less clear. This makes it quite difficult to see consistency and relevance of frequency selections across tasks.

**We did not categorize word frequency into high versus low. Instead, we analyzed word frequency as a continuous variable in our modeling analyses. There are two reasons for not applying a threshold. First, word frequency is inherently a continuous variable, not a categorical one. Dichotomization of continuous variables often leads to loss of power (Cohen, 1983, *Applied Psychological Measurement*). Second, while some researchers use thresholds to treat frequency categorically, this approach was not suitable for our study. We analyzed Japanese items using Japanese word frequency data and English items using English word frequency data. These frequency data, sourced from Twitter, blogs, and newspapers (per million words) [47], are language specific. Therefore, we treated word frequency as a continuous variable in the analyses.**

>>10. Inconsistent definition of ‘difficulty’ and ‘creativity’ makes it less clear how each term is unique in its own way in terms of cognitive load. The overlap between the two (difficulty =problem solving and creativity=semantic distance) is not detailed.

>>How was it ensured that participants see them as separate constructs during rating tasks?
**We conducted two rating tasks separately. We added an explanation in the procedure on page 12.**

**Two rating tasks were conducted separately. First, participants were asked to join a practice session for the difficulty rating task. After the practice session, they were asked to complete the difficulty rating task. Then, participants were guided to the creativity rating task starting with a practice session and followed by a main session.**

**However, although we assumed that difficulty and creativity are unique with each other, our correlation analysis revealed that they were highly correlated (r = .95, p < .001). Therefore, we mentioned in implications that those two ratios were basically reflecting the same aspect in reasoning or might be posited in a hierarchical manner.**

**(p.33) …those two ratios were basically reflecting the same aspect of the analogical reasoning problems. Another explanation is that difficulty and creativity are two different aspects but are posited in a hierarchical manner in which difficulty is considered the superordinate of creativity. Therefore, participants might have found a problem difficult, and that sense of difficulty made them think the problem was creative. Studies on related factors on analogical reasoning problems have started only recently and the relations between these factors have not been fully investigated yet. Multivariate analysis with those factors to investigate the relations between them is encouraged for future studies.**

>>11. Cultural considerations have been shown to some extent but not adequately accounted for in L1 and L2 processing. The example of ‘filament’ used from previous studies may be unfamiliar due to cultural differences and not linguistic differences. This makes the study’s assumption about word frequency as a purely linguistic factor problematic.

**Cultural aspects are not our research scope. However, we used Japanese word frequency for L1 stimuli and English for L2 stimuli. Although it is not a perfect way to consider all cultural aspects, if there is a culturally familiar concept/entity in Japanese but not in English (or vice-versa), that should be reflected in the form of different frequencies of the words for the concepts in different languages.**

>>12. The methodology does not address issues of cognitive load if any, like attentional demands and anxiety in processing analogies in L2.

**We did not consider attentional demands and anxiety in processing analogies in L2. However, we believe that the result on word frequency effects can explain an aspect of cognitive load due to the use of unfamiliar languages in reasoning. To highlight the reason why we included word frequency in the analysis, we modified the paragraph on page 8 (as we responded to comment #6).**

**Also, we highlighted the findings on word frequency effects as a possible explanation for an aspect of the cognitive load in L2 reasoning.**

**(p.30) ... Taken together, the results of the present study explain that one of the aspects of cognitive load in L2 reasoning is most likely related to the inefficiency of word recognition, and that makes participants focus on reading words but not processing relations of words.**

>>13. It is not clear from the manuscript how the 100 analogical problems were ordered or presented. If this is present in the study, it needs to be clear in the methodology.

Thank you for pointing out. We added an explanation in the methodology on page 13.

**(p.13) The order of 100 problems and the positions of multiple choices were randomized.**

Overall, the authors need to improve the manuscript by streamlining the background of the study and rearranging the flow of information in the manuscript for consistency. They also need to critically engage the literature and clarify the hypothesis. It should be clear in the methodology section how control over word selection, clarity in defining predictors, addressing cultural factors and cognitive load was done. This information should not be nested in the analysis but stand out in the methodology. Once these issues are addressed, the study will be firmed and improved.

Reviewer #3: The manuscript attempts to make a contribution to the existing literature on analogical problem-solving among bilinguals. There is indeed a growing body of research in this area. In this manuscript, the authors conducted two experiments among bilinguals with the first experiment requiring them to complete behavioural tasks in their native language (Japanese) while the second experiment presented a series of tasks for them to complete in their second language (English). The manuscript was largely well written except that I have some few concerns which I believe when addressed will substantially improve its suitability for publication. These issues are catalogued below;

>>1. Clearly numerous studies have been conducted on this subject. So, it is not clear what specific knowledge will be derived from this study. The study failed to highlight the novelty of the research conducted. What specific knowledge gap does this study seek to address? This should be emphasized in the early part of the introduction.

**Thank you for your comment. Although “PLOS ONE accepts scientifically rigorous research, regardless of novelty” (**[**https://everyone.plos.org/about-plos-one/**](https://everyone.plos.org/about-plos-one/)**), we do agree that it is better to highlight the novelty. We explained a gap between the results from the previous studies and what should be further investigated.**

**(p.3) These prior studies focused on the accuracy of problem solving in L2, rather than investigating the underlying mechanisms of the reasoning process itself. In analogical reasoning, knowledge transfer between domains occurs based on similarities [3]. However, how this similarity information is transferred in L2 remains unexplored. In addition, simply investigating the accuracy of analogical reasoning is not sufficient to fully understand at what point the differences between L1 and L2 reasoning processes occur.**

>>2. The study failed to include any theory in the literature review.

**We introduced a general framework proposed by Huettig et al. [26]. However, the discussion of the framework seemed to be undermined by the complexity at the beginning of the introduction. Therefore, we revised the paragraphs on page 2. Also, we added the following explanation to better highlight the novelty of this research:**

**(p.6) Although visual information processing with lexical input may offer valuable insights into the area of verbal analogical reasoning in L1 and L2, these two lines of research have been studied separately. Integrating knowledge from these different topics helps investigate how perceptual information is processed during verbal analogical reasoning. …**

**The present study offers new insights into both bilingual and psychological studies by investigating the mechanism of problem solving in L1 and L2. Specifically, the present study highlights the types of similarity and language conditions through the three research questions below.**

>>3. On methodology, in both experiments, participants completed the tasks online. It is not clear at what time, location and devices (PCs, mobile phones, tablets?) were used to complete the tasks. Since this is an experiment, what control measures put in place to eliminate potential extraneous variables that could impact the results?

**We clarified the explanation in the method on page 11.**

**(p.11) Participants were asked to find a quiet place with a stable internet connection and to use PC with a mouse. Please also see our response to Reviewer 2 above.**

>>4. Moreover, the sample size is very small. What is the justification for the use of this small sample size? I recommend a larger sample size.

**The number of items used in our study largely exceeds that of previous research using a few paragraphs (e.g., Wakebe et al., 2015: 31-36 participants in each group and one data point per one participant; Francis, 1999: 33-76 participants in each group and one data point per one participant), with each participant responding to 100 items. We also conducted a post-hoc power analysis for the Accuracy and RT models, referring to Faul et al. (2009) and using G*Power 3.1.9.2. Following their approach, we created two models: one with a set of fixed effects and one without. This approach allows us to perform an *F*-test on the variance explained by the fixed effects (difficulty, category, language, trial count and word frequency). The final model for accuracy achieved a power (1-β err prob) of 0.99, while the RT model reached a power of 0.71. Although 0.71 is lower than the expected standard (0.8), at least this must be better than the previous studies.**

>>5. The presentation of the results in the text largely does not follow the APA format. I see that it is mostly the p-values that are stated. This should be more than that.

**Because the tables and the figures provide more information than p-values, we avoided redundant descriptions (e.g., including slopes, t-values, confidence intervals) in the main texts.**

Reviewer #4: This paper provides an insightful exploration of analogical reasoning and the differences between processing analogies in first (L1) and second (L2) languages. Although I come from a background in mathematics and computational social behavior, I found the manuscript accessible and well-written, allowing me to easily follow the arguments and conclusions. The authors clearly cover relevant prior literature, provide strong motivation for their study, and pose interesting research questions.

**Thank you for your careful reading. We are happy to hear this positive comment from an expert from a different field.**

The paper focuses on whether language differences (L1 vs. L2) influence reaction times and accuracy in analogical reasoning. Additionally, the authors examine the extent to which perceptual and relational similarities affect these two metrics and whether there is a significant interaction between similarity types and languages. The paper approaches these questions by conducting two experiments with bilingual speakers of Japanese (L1) and English (L2).

The results show that analogical reasoning in L1 (Japanese) leads to both faster response times and higher accuracies compared to L2 (English). This suggests that participants are more efficient in their native language when performing cognitive tasks that require complex reasoning. The authors also find that perceptual similarity (e.g., object shape) is particularly challenging when processed in L2, likely due to the increased cognitive load involved in mentally simulating visual features in a foreign language.

As I already mentioned, I am not in this field, so I apologize if my suggestions and questions are not fully relevant. I have some questions and suggestions for the authors:

>>1. The sample sizes of 42 and 41 seem relatively small. Was a power analysis conducted to justify the number of participants?

**We conducted a post-hoc power analysis for the Accuracy and RT models, referring to Faul et al. (2009) and using G*Power 3.1.9.2. Following their approach, we created two models: one with a set of fixed effects and one without. This approach allows us to perform an *F*-test on the variance explained by the fixed effects (difficulty, category, language, trial count and word frequency). The final model for accuracy achieved a power (1-β err prob) of 0.99, while the RT model reached a power of 0.71. Although 0.71 is lower than the expected standard (0.8), the number of items used in our study largely exceeds that of previous research using a few paragraphs (e.g., Wakebe et al., 2015: 31-36 participants in each group and one data point per one participant; Francis, 1999: 33-76 participants in each group and one data point per one participant), with each participant responding to 100 items.**

>>2. Some sentences in the abstract, such as “In this experiment, participants also rated the difficulty and creativity of problems” and “A significant interaction was found between languages (L1/L2) and the category of problems,” state facts but do not inform the reader of the actual results. For example, the fact that perceptual similarity is particularly challenging when processed in L2 is not mentioned in the abstract. I believe the current abstract does not do justice to the results, and I would recommend the authors revisit it.

**We appreciate your comment. We revised the abstract and mentioned that we investigated the similarities in analogical reasoning as below.**

**A significant interaction was found between languages (L1/L2) and the category of problems which indicates that finding a perceptual similarity (e.g., the shape image of word concepts) with verbal stimuli in L2 is more challenging than in L1. Moreover, our results on response times indicated that processing relations between words would be carried out in L1 without any specific instruction while it would not be completed in L2 possibly due to the cognitive demand related to lexical processing.**

>>3. The authors begin the abstract by stating, “Analogical reasoning plays a significant role in language learning.” Could the authors elaborate on the implications of their findings, such as how they apply to language learning, for example in educational settings or in designing better tools for bilingual individuals? This could be addressed in the conclusion.

**Thank you for your comment. In conclusion, we added a piece of possible instructional advice to teachers and instructors following our findings.**

**(p.35)** **Taking them into account, giving L2 learners efficient time to process words and explicitly instructing them to understand the relations of words when making analogies is expected in an educational setting.**

**Also, as you pointed out, the abstract started with motivation in learning but did not end with the same focus. To be consistent, we revised the abstract.**

>>4. One disadvantage of the paper is its length. While the authors have organized it well with sections and subsections that clearly convey specific topics and summarize the results as they go, it remains a lengthy text without much visual aid. I suggest adding a conceptual figure summarizing the experiments. For instance, a visual could show that there are two language groups, the A:B:C format (number of questions), the different categories being tested, and the fact that you are measuring both accuracy and response time. Please note that this is simply a recommendation.

**As advised by other reviewers, we removed the explanation about Ray problems and Lightbulb problems and added them in the notes. Also, we revised the entire section on page 2. Also, we removed the explanation of the word association test and put it in the appendix. By doing these, we shorten the manuscript by 12663 words (from13567 words to 12663 words).**
